# Oral lymphoepithelial cyst: A clinicopathological study of 26 cases and review of the literature

**DOI:** 10.4317/jced.54072

**Published:** 2017-08-01

**Authors:** Maria Sykara, Panagiotis Ntovas, Eleni-Marina Kalogirou, Konstantinos I. Tosios, Alexandra Sklavounou

**Affiliations:** 1 DDS, Faculty of Dentistry, National and Kapodistrian University of Athens, Athens, Greece; 2 DDS, Postgraduate Student, Department of Operative Dentistry, Faculty of Dentistry, National and Kapodistrian University of Athens, Athens, Greece; 3 DDS, MSc, PhD Candidate, Department of Oral Medicine and Pathology, Faculty of Dentistry, National and Kapodistrian University of Athens, Athens, Greece; 4 DDS, PhD, Assistant Professor, Department of Oral Medicine and Pathology, Faculty of Dentistry, National and Kapodistrian University of Athens, Athens, Greece; 5 DDS, MSc, PhD, Professor, Department of Oral Medicine and Pathology, Faculty of Dentistry, National and Kapodistrian University of Athens, Athens, Greece

## Abstract

**Introduction:**

Τo describe the clinicopathological features of 26 oral lymphoepithelial cysts (LECs) and review the literature.

**Material and Methods:**

Twenty-six cases of oral LECs diagnosed during a 37-year period were retrospectively collected. The patients’ gender and age, as well as the main clinical features of the cysts were retrieved from the requisition forms. The main microscopic features were recorded after reevaluation of all cases. Pubmed and Google Scholar electronic databases were searched with the key word “oral LEC”. Inclusion criteria were the microscopic confirmation of LEC diagnosis and the report at least two of three main clinical features (gender, age and cyst’s location).

**Results:**

The 26 oral LECs represented 0.08% of 31,564 biopsies accessioned during the study period. They affected 25 patients, 14 females and 11 males with a mean age of 33.04±9.81 years. They appeared as smooth (92%) nodules, with soft (24%) or firm (76%) consistency and normal (28%), yellow to normal (20%), yellow (32%) or white (20%) hue, in the tongue (69.23%) or the floor of mouth (30.77%). They were covered by parakeratinized squamous (92.31%) or non-keratinized (7.69%) epithelium and contained desquamated epithelial cells, amorphous eosinophilic material and/or inflammatory cells (100%). The lymphoid tissue surrounded the cystic cavity partially (34.62%) or completely (65.38%), often in a follicular pattern with prominent germinal centers (53.85%). Literature review yielded 316 cases of oral LECs derived from 25 case reports, 3 case studies/retrospective studies with detailed information for each case and 7 studies with summarized data.

**Conclusions:**

Oral LEC is a pathologic entity with discrete clinical presentation that is, however, commonly misdiagnosed in clinical practice as other, mostly benign, entities. Its pathogenesis remains obscure, as its clinicopathologic features are consistent with both theories suggested up to date.

** Key words:**Oral lymphoepithelial cyst; developmental cyst; non odontogenic cyst; lymphoid tissue; oral tonsil.

## Introduction

The oral lymphoepithelial cyst (LEC) is a rare, soft-tissue, developmental cyst (1,2), first described by Gold (3) in 1962 as “branchial cleft cyst”. The name LEC that was in use for the description of branchial cysts of the neck (4,5) was proposed by Bhaskar (6) in 1966. Other names applied were “branchial cleft cyst” (7,8), “branchiogenic cyst” (9) or “tonsillar pseudocyst”(10).

LECs arise in various organs, i.e. pancreas (11), stomach (12), thyroid (13), esophagus (14) and mediastinum (15). In the head and neck area it is most common in the lateral cervical region and the parotid glands, with LECs of major salivary glands associated with the human immunodeficiency virus (4,16). Oral LECs usually present in the floor of mouth or the lateral margin of tongue, as painless nodules of normal-yellow to white color and soft to firm consistency, measuring less than 1cm (17-21). Microscopically, the cystic cavity is lined by stratified squamous or/and pseudostratified columnar epithelium and contains desquamated epithelial cells and inflammatory cells. The fibrous connective tissue wall of the cyst is surrounded by lymphoid tissue, usually with a follicular pattern (6,17,18,20). The pathogenesis of intraoral LEC has not been settled (3,10,22).

The objective of the present study is to describe the clinicopathological features of 26 oral LECs and review the pertinent literature with emphasis on its histopathogenesis.

## Material and Methods

All cases of LEC diagnosed in the pathology laboratory of the Department of Oral Medicine and Pathology between 1980 and 2016 were retrospectively collected. LECs of the major salivary glands were excluded. Patients’ gender and age, location, clinical features (color, consistency, surface texture and maximum dimension), symptoms and duration of the lesion before diagnosis, as well as clinical diagnosis were collected from the requisition forms. The main clinical features of the cases studied are summarized in  Table 1. The histopathological features studied were type of the lining epithelium, cystic content, pattern of lymphoid tissue, type of adjacent anatomic structures and overlying mucosa. All patients at the time of their initial examination gave written consent for the future use of their data for study. The study was approved by the Research Ethics Committee (NKUOA code number 310).

**Table 1:**
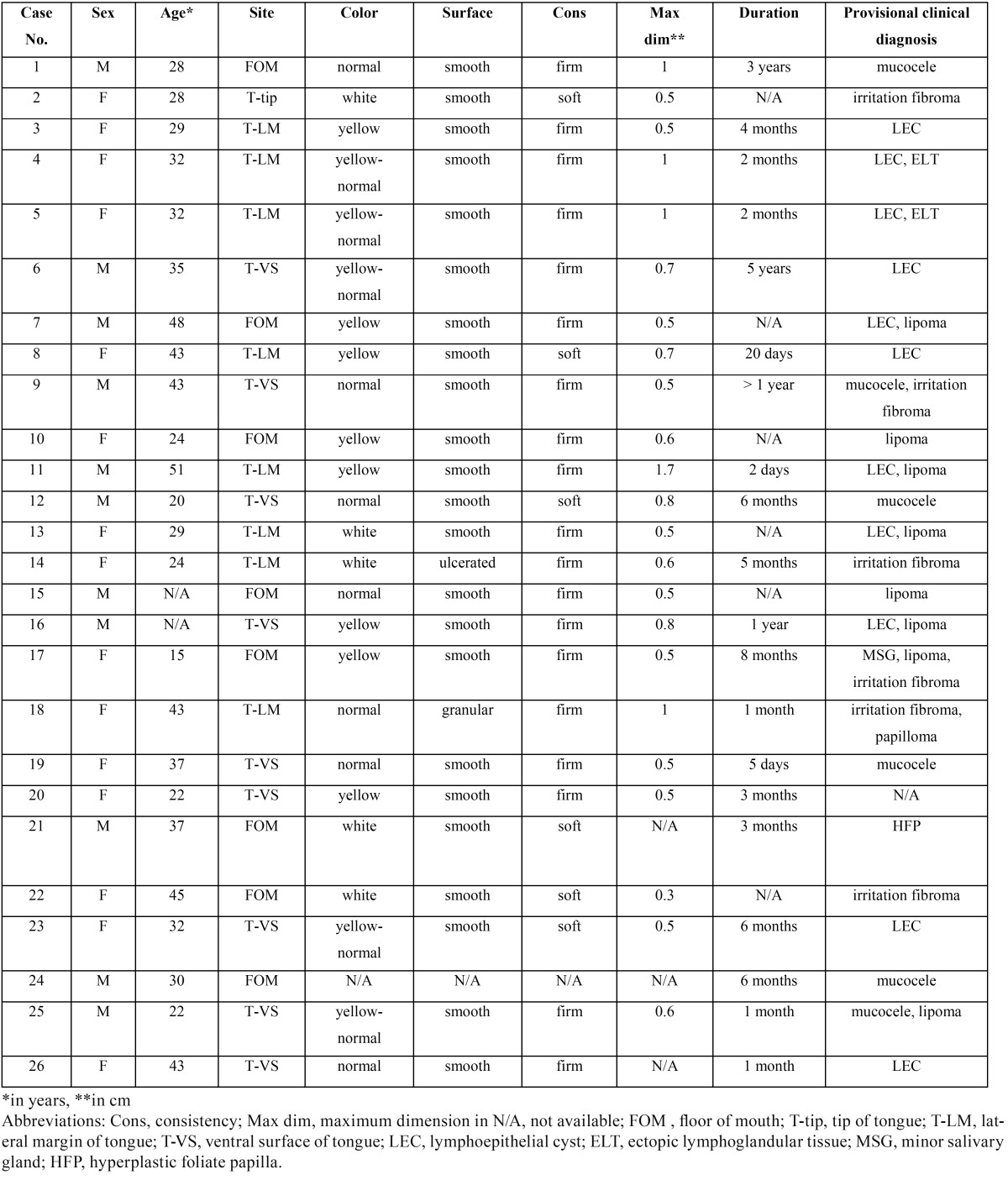
Demographics and clinical characteristics of the 26 oral lymphoepithelial cysts.

Pubmed and Google Scholar electronic databases were searched on April 2017 with the key word “oral lymphoepithelial cyst” and the pertinent literature was collected. Studies included were those where the diagnosis had been confirmed by microscopic examination and at least two of three main clinical features (gender, age and location of the cyst) were reported. Cases located on the palatine tonsils, oropharynx or jawbones were excluded from evaluation.

## Results

-Demographics

Twenty six oral LECs in 25 patients were found (a 32-year-old woman presented two adjacent intraoral LECs) among 31,564 biopsies accessioned during the study period, representing 0.08%. Fourteen patients were females (56%) and 11 males (44%), with a female to male ratio of 1:0.79. Age was known in 23 patients and ranged from 15 to 51 years (mean age=33.04±9.81 years). Most LECs were found in adults in 3rd (39.13%), 4th (26.09%) or 5th (26.09%) decade of life, only 1 case considered an adolescent.

-Clinical features

**Figure 1:**
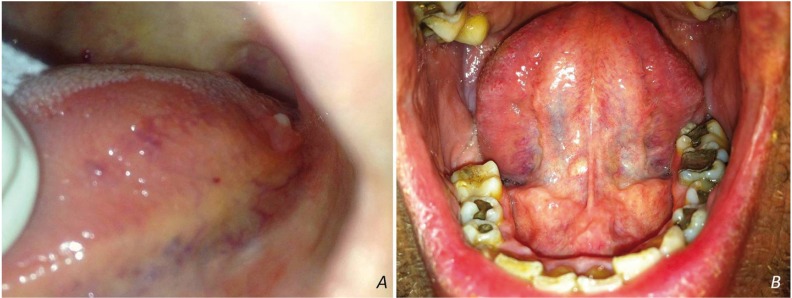
Yellow-white nodules on the posterior lateral margin of the tongue (A) and floor of mouth (B).

LECs appeared as nodules, covered by normal (28%), yellow to normal (20%), yellow (32%) or white (20%) mucosa. The surface was smooth (92%) and rarely ulcerated (4%) or granular (4%). Consistency was reported as soft (24%) or firm (76%). Clinical information was missing in one case. The majority of lesions (18 cases, 78.26%) measured less than 1cm in greatest dimension (range=0.3-1.7cm, mean dimension=0.69±0.3cm). Most LECs were located on the tongue (18 cases, 69.23%), in particular the ventral surface (9 cases, 50%), the lateral margins (8 cases, 44.44%, Fig. 1A), or the tip (1 case, 5.56%), followed by the floor of mouth (8 cases, 30.77%, Fig. 1B). The two adjacent lesions in a 32-year-old female were located on the posterior lateral margin of the tongue and were clinically identical.

Twenty LECs were noticed 2 days to 5 years before biopsy and 16 of them (80%) were excised during the first year. Oral LEC was the most common clinical diagnosis, reported in almost half of cases (11/26, 42.3%). Other clinical diagnoses included lipoma, mucous extravasation cyst (mucocele), irritation fibroma, squamous papilloma, hyperplastic foliate papilla, ectopic lymphoglandular tissue and minor salivary gland. All lesions were asymptomatic and surgically excised under local anesthesia.

-Histopathological features

**Figure 2:**
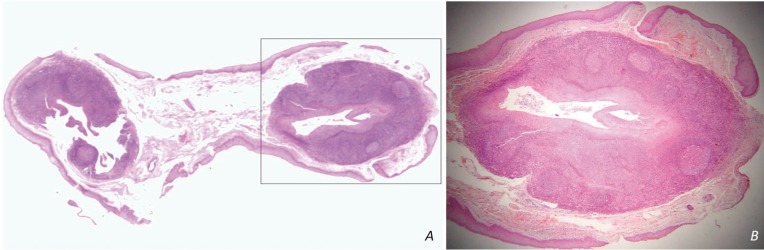
A) Two adjacent LECs from the lateral lingual margin with similar histopathological features. B) The cystic cavity is lined by parakeratinized stratified squamous epithelium with prominent hyperplasia of the parakeratin layer [hematoxylin and eosin stain; original magnifications (A) x1, (B) x25].

**Figure 3:**
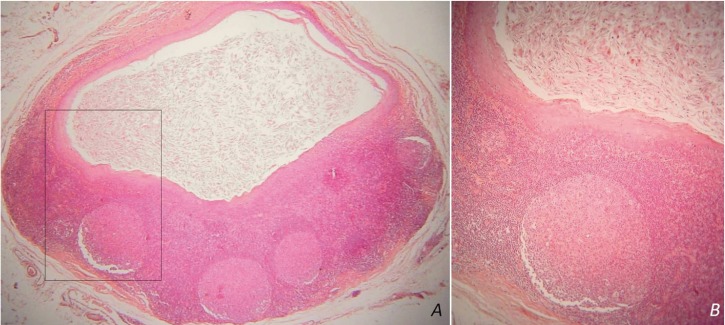
Lymphoid tissue surrounds the cystic cavity in a follicular pattern with prominent germinal centers. The cystic lumen contains desquamated epithelial cells, amorphous eosinophilic material and inflammatory cells [hematoxylin and eosin stain; original magnifications (A) x25, (B) x100].

 Table 2 summarizes the main histopathological data of the cases studied. The two adjacent LECs showed similar histopathological features (fig. 2A). In 24 cases (92.31%) the cystic epithelium was parakeratinized and in 17 of them (70.83%) prominent hyperplasia of the parakeratin layer was noticed (fig. 2B). In one of those cases transition from squamous to pseudostratified columnar epithelium was also seen. In 2/26 cases (7.69%) the cyst was lined by non-keratinized squamous epithelium with a superficial layer of cuboidal cells. Mucous cells or goblet cells were not found. The inflammatory cells infiltrated focally the cystic epithelium in most cases. The lymphoid tissue surrounded the cystic cavity partially (9 cases, 34.62%) or completely (17 cases, 65.38%) and was arranged in a follicular pattern with prominent germinal centers (14 cases, 53.85%, Fig. 3A,B). The cystic lumen in all cases was filled with desquamated epithelial cells, amorphous eosinophilic material and/or polymorphonuclear leukocytes, lymphocytes and plasma cells (Fig. 3A,B). The cystic wall consisted of non inflammatory, loose to dense fibrous connective tissue and exhibited areas of epithelial islands and minor salivary glands in one and six cases, respectively.

**Table 2:**
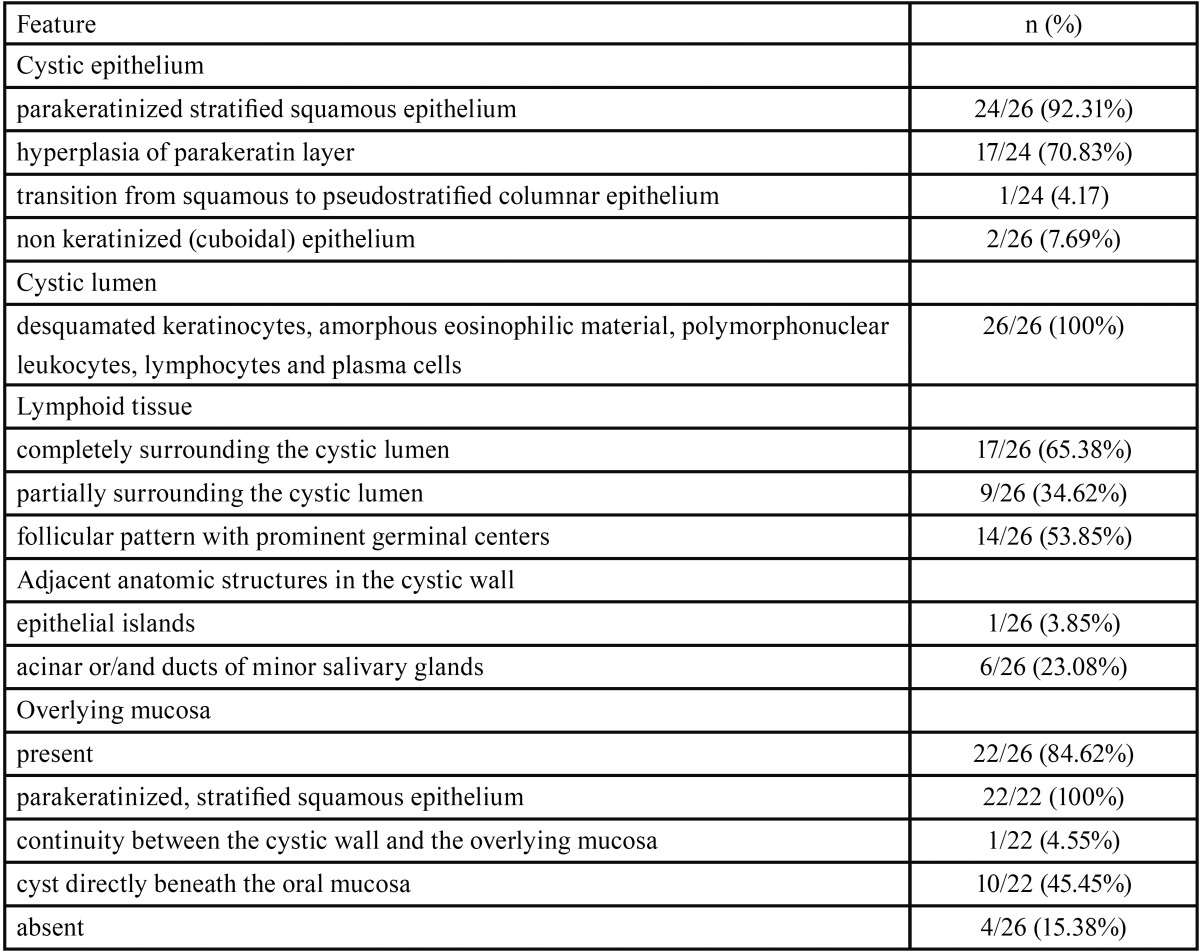
Histopathological features of the 26 oral lymphoepithelial cysts.

The overlying mucosa was present in the histopathological sections of 22 cases (84.62%) and was covered by parakeratinized, stratified squamous epithelium. In 10 cases the cyst was located directly beneath the oral mucosa. Continuity of the cystic epithelium and the overlying mucosa was noticed in one case.

-Literature review

Forty-five papers were retrieved; 10 case-reports were excluded, 7 due to inability to access full-text (23-29) and 3 due to non-intraoral location (30-32). Only two cases from a report of multiple LECs in the same patient (33) were included, as the rest lacked histopathological confirmation, while for the same reason another case (34) included in previous reviews (35,36) was omitted. In total our review included 316 cases of oral LEC ( Table 3, Table 3 continue) derived from 25 case reports (3,7-9,19,21,22,33,35,37-52), 3 short case studies/retrospective studies (17,20,53) with detailed information for each case, as well as 7 studies (1,2,6,10,18,36,54) with summarized sample data. In three of the latter studies (1,2,36) there were 8 cases in the palatine tonsil or the oropharynx that could not be excluded, due to the way the data were reported.

**Table 3:**
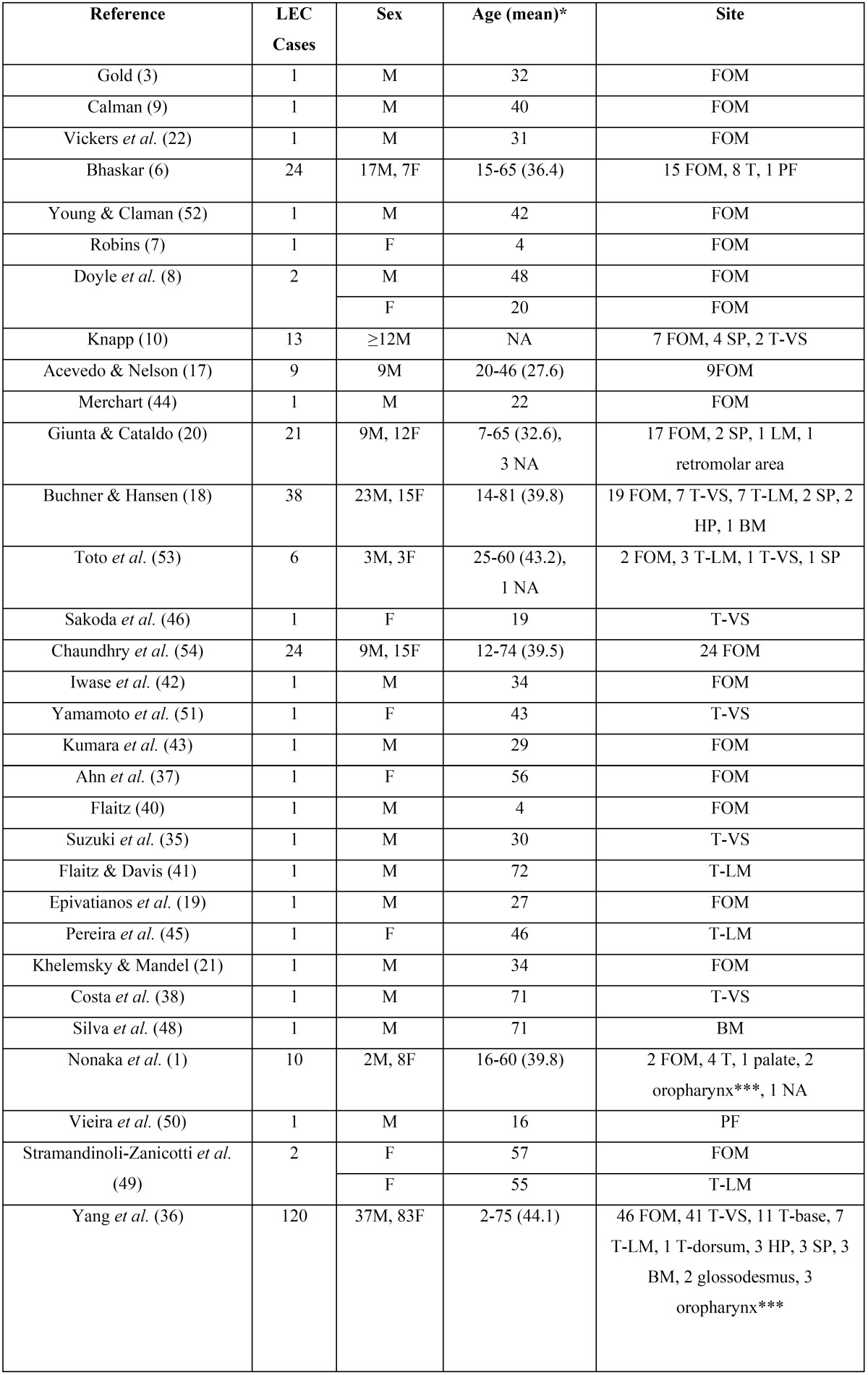
Review of the demographical features, location, maximum dimension and duration of 316 previously reported cases of oral LEC.

**Table 3 continue:**
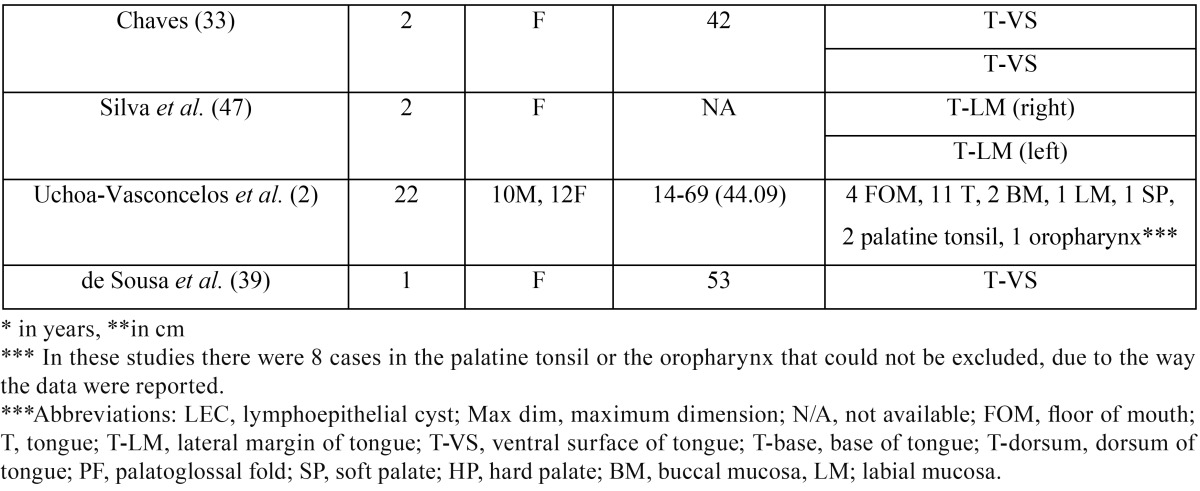
Review of the demographical features, location, maximum dimension and duration of 316 previously reported cases of oral LEC.

## Discussion

The 26 LEC cases presented herein represent less than 0.1% of all biopsies accessioned in a single oral pathology laboratory during a 37-year period, while in two previous studies they respective figures were 0.11% in 53 years (2) and 0.1% in 40 years (1). The asymptomatic nature of LECs that make their recognition mostly an incidental finding (3,9,19,39,49), as well as their clinical misdiagnosis as normal anatomic structures, e.g. foliate papillae (53) may account for the rarity of recorded cases.

In this study oral LEC showed a slight female predilection (female to male ratio 1:0.79), while in 316 previously reported cases (table 3) there is considerable no predilection (female to male ratio 1:0.9). Previously reported oral LECs showed a broad age distribution (2-81 years), but they were more common in 3rd to 5th decades of life (1,2,6,18,20,36,54), which is in accordance to the present study. Children and adolescents are rarely affected, with approximately 14 cases reported (1,2,6,7,10,18,20,36,40,50), while there is a unique case of oral LECs in a mother and her daughter (20).

Lingual LECs represented more than two thirds of the cases presented herein, with the remaining cases located on the floor of mouth. In contrast, among 316 cases included in our review ( Table 3), more cases (n=160, 50.63%) were located in the floor of mouth compared to the tongue (n=114, 36.08%), followed in descending order by the soft palate, buccal mucosa, hard palate, labial mucosa, palatoglossal fold, glossodesmus and retromolar area. There is no other report of two clinically identical, adjacent LECs, while there are some scarce reports of multiple LECs in the same patient (33,47). The coexistence of oral LEC with an epithelial inclusion cyst has been also reported (19,37).

Oral LEC manifested as a soft to firm, well defined submucosal nodule, covered by mucosa with normal, yellow or white hue, as in most previous studies (9,10,17-19-21,22,33,35-38,40). The yellowish color has been attributed to the keratin or the purulent content that is commonly found within the cyst’s lumen (10,20). Oral LECs with transparent, translucent, purple or gray hue have been rarely recorded (17,36). The surface of LEC is generally smooth (3,9,19,21,22,33,37,39,40,45), but may be ulcerated (35) as seen in one of our cases, probably due to secondary trauma. They are predominantly less than 1cm (6,17-21,33,35,36,38,39,42,45), may be present for several days to years (2,6,7,17-19,21,35-37,40,42) and are generally painless (3,9,17-22,33,36-39,42,45,46). Symptoms, such as pain or discomfort in swallowing may be occasionally reported, for example in case of large (7) or ulcerated (35) lesions.

In most cases LECs are clinically misdiagnosed as mucus cyst (9,18-20,36,37,46,48,51-53), with the list of clinical diagnoses also including normal anatomic structures, such as foliate papilla, sebaceous gland, sublingual gland or lymphoid tissue (18,20,36,50,53), as well as pathological entities, including lipoma, fibroma, inflammatory fibrous hyperplasia, squamous papi-lloma, granular cell tumor, dermoid cyst, inclusion cyst, abscess, sialolithiasis, sialadenitis, nevus and leukoplakia (8,18,20,35,36,38,48-50,53). The high accuracy of clinical diagnosis in the present study may be associated with the location of all cases in typical anatomical sites, e.g. tongue and floor of mouth.

The microscopic features of LEC are described is studies performed more than three decades ago (6,10,17,18,20,53). The sample size of the present case series is the largest, after the study by Buchner and Hansen (18) published 37 years ago. Oral LECs are predominantly lined by thin (7,9,18,20,50), parakeratinized stratified squamous epithelium (6,17-20,33,35,37-40,42,45,46), with flattened rete pegs (3,6,9,17-19,21,22,35,39,46), probably due to the pressure exerted by the surrounding lymphoid tissue (35). The lining epithelium may be, also, orthokeratinized (18) or non-keratinized (22,52) stratified squamous, pseudostratified columnar (7,18,20,48,50) or ciliated columnar (20), and contain mucous/goblet cells (18,20,40). It is, occasionally, infiltrated by lymphocytes or polymorphonuclear leukocytes (7,9,42,50,52). The cystic cavity is occupied by desquamated epithelial cells, keratin or amorphous eosinophilic material, and inflammatory cells (6,9,10,17-21,35,37,42,46,49). The lymphoid tissue surrounds the entire cyst or part of it (18,20,42), often in a follicular arrangement with prominent germinal centers (3,6,18-22,33,37,40,42,45,49). Epithelial islands (6,17), mucous glands or duct-likes structures (6,18,21,46), microorganisms (18,53) and calcified material (18) may be seen in the lymphoid aggregates or the surrounding fibrous connective tissue. Most of those features were seen in our cases, where the lining epithelium was predominantly hyperparakeratinized stratified squamous.

Considering the pathogenesis of oral LECs, there are two competing theories. The theory of embryologic entrapment or epit-helium enclavement supports that epithelium becomes entrapped in the oral mucosal lymphoid tissue and following proliferation due to trauma or chronic inflammation forms the cyst (3,6,22). The entrapped epithelium may be remnants of the branchial arches that are found in the floor of mouth, a common localization of LEC (3). According to this theory LEC is a developmental lesion, but this is not consistent with the wide age distribution of oral LEC (46). The epithelium may also originate from the oral mucosal (22) or the salivary ducts (6,22), as epithelial islands and duct-like structures may be identified within the lymphoid tissue or in close proximity with it (6,17,18,21,46) and may undergo cystic changes (6). The presence of columnar epithelial lining observed in several LECs and the continuity of the cystic epithelium with salivary gland ducts favor the latter hypothesis (20,21,53). In support of this theory is also the finding of in vitro studies, where surgical transplantation of buccal epithelium in lymph nodes of hamsters induced the formation of cysts (55).

According to the obstruction theory, a traumatic event or a microbial invasion accompanied by inflammatory reaction may stimulate the parakeratinization of the crypt’s non keratinized epithelium (17,18,20,36). With incomplete desquamation, the parakeratinized squamous epithelial cells along with bacteria and purulent material accumulate and obstruct the crypt’s orifice, resulting in the formation of LEC (10,18-20). According to this theory, LEC is a pseudocyst LEC. This theory is supported by the most common presence of oral lymphoid tissue (“oral tonsils”) in the floor of mouth, tongue and soft palate (10), i.e. the three most frequent sites of intraoral; the presence of inflammatory infiltration of the LEC epithelium; and the continuity or the close proximity of the cystic epithelium with the superficial oral mucosa (19,21,46,53). Continuity, though, may disappear in advanced developmental stages of oral LEC (18) or may not be detected in sections evaluated; thus, lack of it does not necessarily reject the latter theory (53). There are cases, however, where pathogenesis in not consistent with either hypothesis (18) or consistent with both hypothesis (19). As in previous reports, the findings of our study may be interpreted by both theories.

Excisional biopsy is usually recommended to confirm the diagnosis of oral LEC. Recurrence has not been reported in a follow up period up to 17 years (36).

In conclusion, oral LEC is a pathologic entity with discrete clinical presentation that is, however, commonly misdiagnosed in clinical practice as other, mostly benign, entities. Its pathogenesis remains obscure, as its clinicopathologic features are consistent with both theories suggested up to date.
